# Nanozyme Aptasensor Array for Predictive Sensing of Virulent and Antibiotic‐Resistant *Staphylococcus Aureus* strains

**DOI:** 10.1002/smll.202512266

**Published:** 2026-02-11

**Authors:** Pabudi Weerathunge, Mahdieh Yazdani, Tarun K. Sharma, Wilson K. M. Wong, Mugdha V. Joglekar, Anandwardhan A. Hardikar, Vincent M. Rotello, Rajesh Ramanathan, Vipul Bansal

**Affiliations:** ^1^ Sir Ian Potter NanoBioSensing Facility NanoBiotechnology Research Laboratory (NBRL) School of Science RMIT University Melbourne Victoria Australia; ^2^ Department of Chemistry University of Massachusetts Amherst Amherst Massachusetts USA; ^3^ Department of Medical Devices National Institute of Pharmaceutical Education and Research Mohali Punjab India; ^4^ School of Medicine Diabetes & Islet Biology Group, Faculty of Health Western Sydney University Campbelltown New South Wales Australia

**Keywords:** aptamers, colorimetric sensor, nanozymes, pattern recognition, predictive diagnostics, *S. aureus*, sensors

## Abstract

*Staphylococcus aureus*, an important human pathogen, is the leading cause of infection‐related death globally. It stands out as the only bacterial pathogen, apart from *Mycobacterium tuberculosis*, responsible for over a million fatalities each year. The emergence of antibiotic‐resistant strains, such as methicillin‐resistant *S. aureus* (MRSA), has created challenges in detecting *S. aureus* infections, as treatment depends on identifying the specific strain causing the infection. This study highlights the use of an array‐based colorimetric aptasensor platform using aptamers, which exhibit specific binding across different *S. aureus* strains. This platform generates unique colorimetric fingerprints for different *S. aureus* strains, thus enabling an unbiased and strain‐specific detection system. The unique signatures arise from differences in the dissociation dynamics of aptamers on the surface of nanozymes. The sensor response was analysed using pattern recognition tools trained on responses from the aptasensor array to identify individual *S. aureus* strains. Furthermore, the sensing platform offers additional functionality by providing information about the virulence factors associated with pathogenicity, such as the presence of markers like Panton‐Valentine leukocidin (*pvl*), which is a marker of increased virulence and sensitivity/resistance to antibiotics. The platform would be capable of recognising previously unencountered *S. aureus* strains, enabling predictive capabilities and utility in clinical diagnostics.

## Graphical abstract

An array‐based nanozyme‐aptamer platform for rapid, colorimetric fingerprinting of Staphylococcus aureus strains is reported. By combining differential aptamer dissociation with pattern‐recognition analysis, the sensor achieves strain‐level discrimination, infers virulence, and predicts the identification of previously unencountered strains, offering a low‐cost strategy for point‐of‐care screening of antibiotic‐resistant bacterial infections.

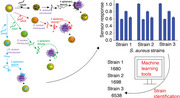

## 1 Introduction

*Staphylococcus aureus* (*S. aureus*), a common Gram‐positive facultative anaerobic bacterium, is the primary cause of infection‐related deaths globally, responsible for over a million yearly fatalities. *S. aureus* is known to cause mild to moderate skin infections in the community or severe infections, especially in hospital settings [1, 2, 3, 4, 5, 6]. This diverse capability of *S. aureus* to cause mild to severe infections is attributed to virulence factors. The most widely accepted treatment for *S. aureus* infections is antibiotic therapy [1, 2, 3, 4, 5, 6]. However, the emergence of antibiotic‐resistant strains, such as methicillin‐resistant *S. aureus* (MRSA) and vancomycin‐resistant *S. aureus* (VRSA) strains, and the presence of *pvl* genes responsible for increased virulence (responsible for the destruction of white blood cells, necrosis, and apoptosis) pose a challenge for effective treatment [7, 8, 9], as the therapeutic approach depends on identifying the specific *S. aureus* strain causing the infection [10, 11, 12, 13, 14, 15]. Although culture techniques are considered the clinical gold standard, long processing times (typically days), personnel costs, and the need for specialised personnel limit their use [16]. In contrast, strain‐level detection has improved significantly with nucleic acid technology (PCR‐based assays) [17]. However, there is still a need for specialised personnel and infrastructure for PCR‐based assays.

Colour‐based detection, such as enzyme‐linked immunosorbent assay (ELISA), is the workhorse of analytical research. ELISA uses natural enzymes to catalyse enzymatic reactions to generate a colour, while specificity is achieved through the use of antibodies [18, 19]. Given the instability and high production costs of natural enzymes, there has been an increasing effort to develop viable alternatives [20, 21]. With the discovery that certain inorganic nanoparticles exhibit enzyme‐mimic catalytic characteristics (nanozymes), there has been significant interest in developing new detection methods in which the organic enzyme is replaced with an inorganic nanozyme (NZ) [20, 21, 22, 23, 24, 25, 26, 27, 28, 29, 30, 31, 32, 33, 34, 35, 36]. Of the several reported NZs, the widespread use of gold nanoparticles (GNPs) is unsurprising owing to their well‐established synthesis, biocompatibility, ease of surface modification, stability in aqueous media, and unique surface plasmon resonance that is influenced by particle size, the distance between particles, and refractive index in the surrounding environment [37, 38]. Similar to the challenges associated with natural enzymes, the use of antibodies also has challenges, including the high cost associated with development, sensitivity to temperature and pH changes, and batch‐to‐batch variability [39, 40]. Single‐stranded nucleic acid molecules, commonly referred to as aptamers, have emerged as a possible alternative for antibodies that can be used as a molecular recognition element (MRE) in sensors [39, 40]. Aptamers fold into complex 3D patterns that enable them to bind to specific targets [39, 40]. Relative to antibodies, aptamers are easy to fabricate and offer low production costs, lower batch‐to‐batch variability, lower immunogenicity, and reversible folding properties [39, 40]. Combining the strength of NZs to generate colour with the high target specificity of aptamers has led to the development of sensors for detecting a wide range of chemical and biological targets [20, 21, 41, 42, 43]. Our group has developed a unique sensing strategy that combines NZs and aptamers [44, 45, 46, 47]. In this strategy, the inherent enzyme‐like catalytic activity of GNP NZs can be blocked by passivating the nanoparticle surface with aptamers raised against a target analyte. The GNP‐aptamer conjugate acts as the sensor probe. When this probe is exposed to a sample containing the target analyte, the higher affinity of aptamers to the target (than the GNP) results in the dissociation of the aptamers from the surface of the GNPs, thereby allowing the GNPs to regain their NZ activity. Using this approach, we have demonstrated the ability to detect targets ranging from small molecules to whole cells [44, 45, 46, 47]. If this approach is used to detect *S. aureus* using aptamers specific for *S. aureus*, in principle, it will result in a positive response, irrespective of the *S. aureus* strain. This implies that the use of a single sensor probe would not be able to achieve strain‐level detection.

**SCHEME 1:**
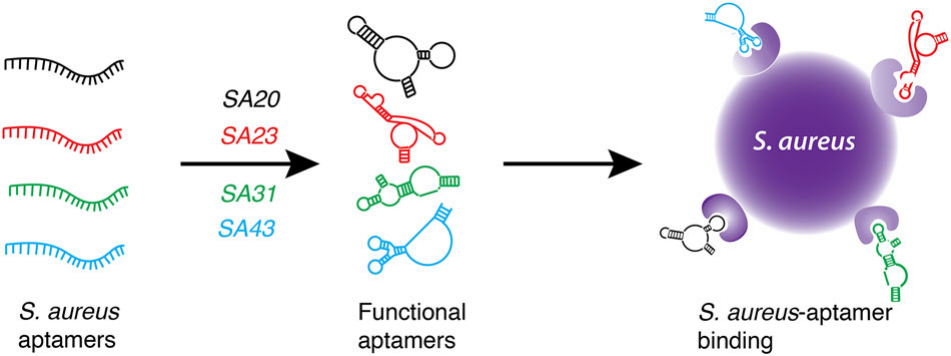
Schematic illustration showing the binding of four aptamers to distinct sites on the surface of *S. aureus*.

Instead, inspired by the high success of array‐based sensing platforms, in this communication, we show a robust colorimetric sensor array where, instead of a single aptamer as the MRE, we use the strength of several aptamers targeting different regions on the surface of *S. aureus* [4] (Scheme 1) to generate a suite of sensor probes. When these probes are exposed to different strains of *S. aureus* independently, the number of aptamers that dissociate from the surface of the NZ would be different. This will generate a unique colorimetric fingerprint that will be specific to each strain. This fingerprint can be analysed using pattern recognition tools that not only identify the specific strain of *S. aureus* but also determine its resistance or susceptibility to antibiotics. This will provide insights into the cell surface markers associated with pathogenicity, offering a comprehensive overview of the strain's virulence characteristics.

## 2 Experimental Section

### 2.1 Synthesis and Characterisation of GNPs

The citrate functionalised GNPs were synthesised based on the Turkevich citrate reduction method and dialysed to remove all unreacted gold ions and citrate. The GNPs were thoroughly characterised using a suite of material characterisation techniques (detailed synthesis and characterisation given in the Supporting Information).

### 2.2 Nanozyme Activity of GNPs

Peroxidase‐like nanozyme activity of the citrate functionalised GNPs was assessed by investigating the ability of the GNPs to oxidise chromogenic substrate 3,3',5,5'‐Tetramethylbenzidine (TMB) to a blue colour product in the presence of H_2_O_2_. Enzyme kinetics parameters, including the Michaelis constant (*K_m_*), maximum velocity of the reaction (*V_max_*), and the turnover number (*K_cat_*), were calculated (detailed in Supporting Information).

### 2.3 Sensor Probe Fabrication

The sensor probes were fabricated by incubating a fixed amount of each aptamer (*SA20*, *SA23*, *SA31* and *SA43*) with the GNPs. The loss of the inherent nanozyme activity of GNPs was assessed by monitoring the oxidation of TMB. A non‐linear least squares fitting was then used to calculate the association constant.

### 2.4 Biosensing Studies

The specificity of the nanozyme array‐based aptasensor was determined by evaluating the recovery of the nanozyme activity (1 min time point) in the presence of seven strains of *S. aureus* and five other pathogens. The recovery was first assessed in buffer conditions. Further studies were carried out in simulated wound fluid (SWF) spiked with different strains of *S. aureus*. All experiments were carried out in 16 replicates, eight replicates used for the training set, and eight replicates used for the test set. Thus, for seven *S. aureus* strains, the colorimetric response was obtained using four sensor probes and eight replicates (7 *S. aureus* strains × 4 sensor probes × 8 replicates).

### 2.5 Statistical Analysis

The sensor response was assessed by hierarchical clustering analysis (HCA) and classical linear discriminant analysis (LDA). HCA was performed on the average absorbance data using OriginPro, using an Euclidean metric with a “*Furthest neighbour*” method of linkage. The data matrix obtained from each experiment was also processed by LDA using SYSTAT software (version 13, SystatSoftware, Richmond, CA, USA). In LDA, the tolerance was set as 0.001 and distances were calculated using Mahalanobis distances. The colorimetric responses were converted to canonical patterns where the ratio of between‐class variance to the within‐class variance was exploited according to the groups that were assigned. Robustness was assessed using leave‐one‐out cross‐validation using jackknife analysis on the training set in SYSTAT. A Naïve Bayes classifier was employed to evaluate the performance of the classification model, where the classification performance was assessed by constructing a confusion matrix.

Randomisation of samples into discovery/test set and validation/training set was performed using the RAND() command in Microsoft Excel (ver. 2016, Microsoft, Redmond, WA, USA). Akaike information criterion (AIC) and receiver operating characteristic (ROC) curve analysis were performed in R software (ver. 3.6.1, R Foundation for Statistical Computing, Vienna, Austria). R package pROC (ver. 1.17.0.1) was used for ROC analysis. R glm() and step() functions were used for AIC analysis. Unknown sample identification was performed by first converting the absorbance values to canonical scores established using the training set. This was followed by the calculation of the Mahalanobis distance for each of the unknowns, and the unknowns were assigned to a particular group based on the shortest distance.

### 2.6 Secondary Structure Prediction and Analysis

Secondary structure prediction and thermodynamic analysis were performed using MFold under ionic conditions representative of the experimental buffer and simulated wound fluid (SWF), with the temperature set to 37°C. Thermodynamic parameters, including Gibbs free energy (ΔG), melting temperature (T_m_), enthalpy (ΔH), and entropy (ΔS), were obtained from the lowest‐energy predicted structures. The effect of SWF on aptamer stability was quantified by calculating the difference in Gibbs free energy. Given the small number of aptamers (*n* = 4), both non‐parametric and parametric statistical analyses were applied to evaluate the consistency of stabilisation across sequences.

## 3 Results and Discussion

**SCHEME 2:**
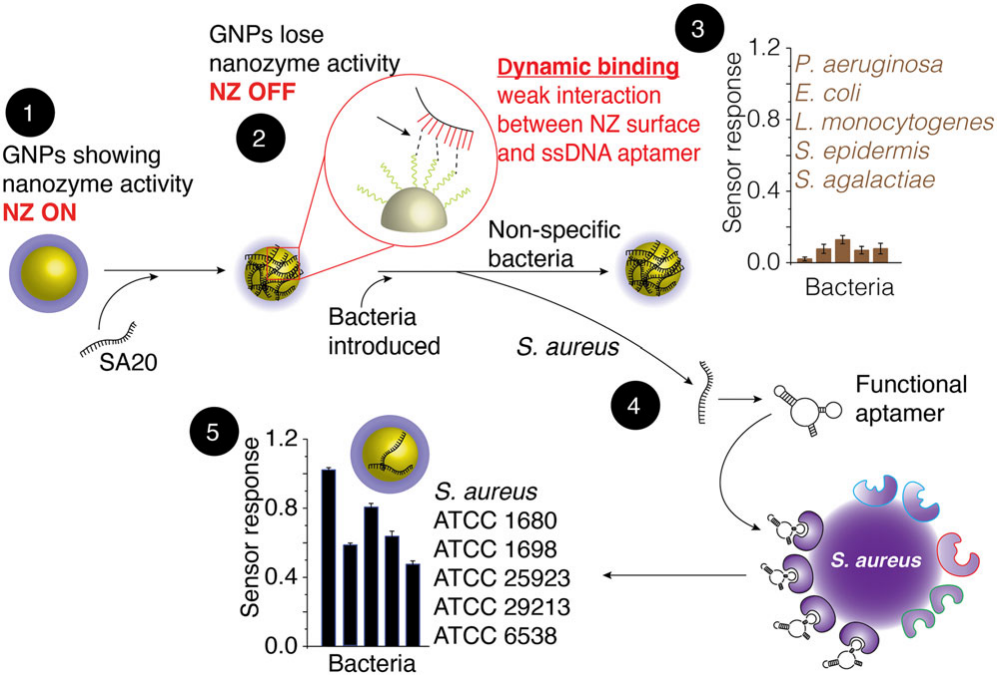
Schematic illustration of the steps involved in detecting *S. aureus* using a single aptamer‐GNP conjugate as the sensor probe. **Step 1** shows the enzyme‐mimicking catalytic activity of GNPs, resulting in the oxidation of TMB substrate (glow around NP). **Step 2** shows that the enzyme‐mimicking catalytic activity of GNPs is lost owing to passivation of the surface with the *SA20* aptamer that specifically binds to *S. aureus* (‘*Sensor Probe*’). **Step 3** shows that when the probe is exposed to non‐specific pathogens, a minimal colour is generated. **Step 4** shows the desorption of aptamers from the surface of the GNPs when the probe is exposed to *S. aureus* and folds into their functional form, binding with *S. aureus*. **Step 5** shows that the surface of GNPs is free to promote the oxidation of TMB, resulting in a colorimetric response in the presence of different *S. aureus* strains.

The principle of the current sensing platform is built around the inherent NZ activity of GNPs to generate a characteristic colour (Scheme 2, Step 1) formed as a result of the oxidation of the peroxidase substrate—TMB (3,3’,5,5’‐Tetramethylbenzidine) in the presence of hydrogen peroxide (H_2_O_2_) (see Figures S1–S4 and Table S1 for the characterisation and enzyme‐like characteristics of GNPs) [28, 44, 46, 47]. To validate whether a single aptamer‐GNP conjugate sensor probe will offer the capability to detect *S. aureus*, the surface of GNPs was first passivised with a single aptamer (*SA20*) specifically raised against *S. aureus*. The non‐covalent binding of the *SA20* aptamer to the GNP resulted in the loss of inherent NZ activity (Scheme 2, Step 2). This GNP‐aptamer conjugate, with no or minimal NZ activity, served as a sensor probe. When this probe was exposed to non‐specific pathogens, the aptamers did not dissociate from the surface of the GNPs, resulting in minimal/no colorimetric sensor response (Scheme 2, Step 3). When the same probe was exposed to *S. aureus* strain 1680, the high affinity of the aptamer to *S. aureus* in comparison to GNPs resulted in the desorption of aptamers from the GNP surface and binding to *S. aureus* (Scheme 2, Step 4). This resulted in a colorimetric response, specifically in the presence of *S. aureus* strain 1680. Interestingly, exposing the probe to other strains of *S. aureus* also resulted in a colorimetric response, albeit with different colorimetric intensities (Scheme 2, Step 5). This is unsurprising as other strains of *S. aureus* would exhibit some degree of surface similarities to strain 1680, which enables the *SA20* aptamer to dissociate from the surface of the GNPs [4].

The strategy of using one aptamer‐GNP conjugate as a sensor probe for bacterial cell detection poses additional challenges in terms of false positives or false negatives owing to (i) antigenic variations in bacterial cells to prevent a host immune response [4], (ii) differential expression of surface virulence factors [9], (iii) expression of different surface molecules based on the growth state [48], and (iv) cell surface mutations [49]. This means that for bacterial detection using the NZ OFF/ON strategy, it is essential to use multiple aptamers that can potentially bind to different regions of the bacterial cell surface. Additionally, it has been recognised that the binding efficiencies of individual aptamers to a target are much lower than those of a pool of aptamers against the target because of similarities in the surface compositions [4]. Therefore, in the current case, if multiple aptamers are used to target different sites on the surface of *S. aureus* (Scheme 1), it may lead to increased selectivity during detection.

**SCHEME 3:**
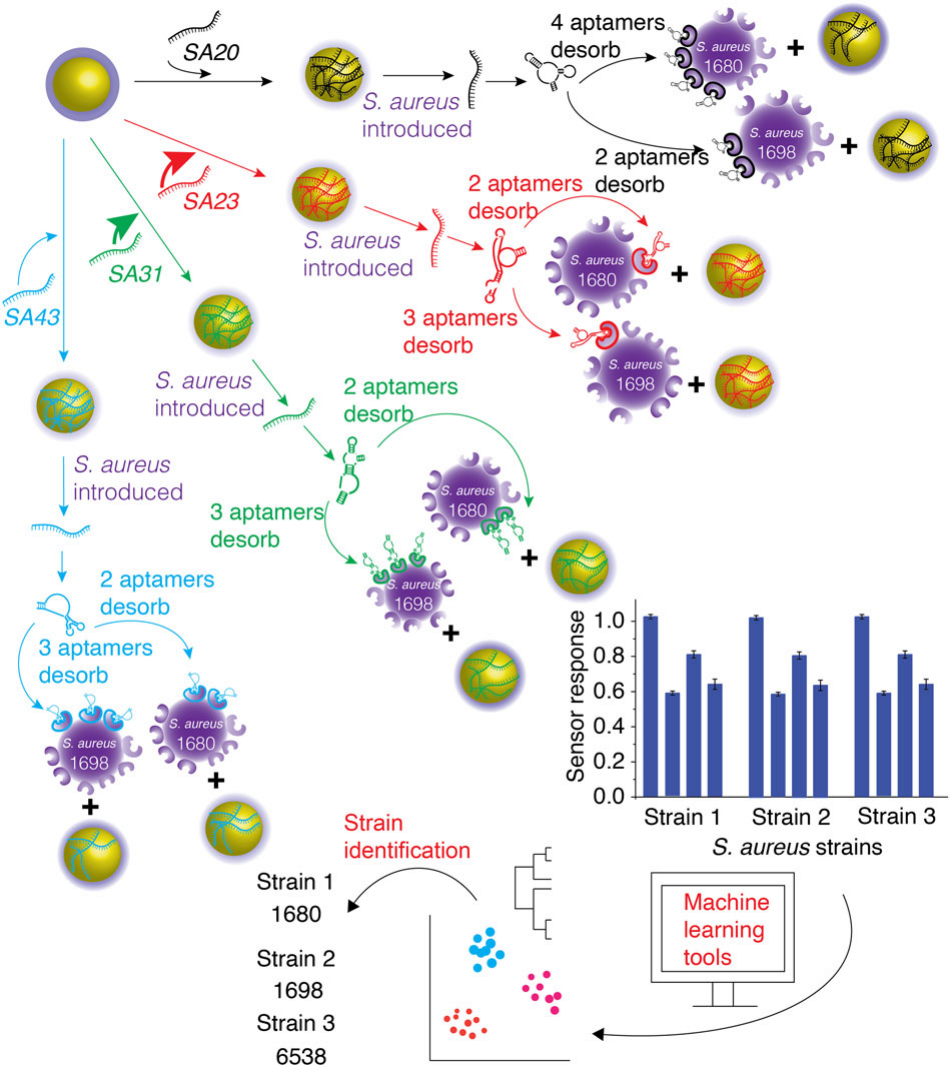
Schematic illustration of the underlying working principle of the array‐based aptasensor used to generate a colorimetric fingerprint for different *S. aureus* strains using multiple aptamer‐GNP conjugates as sensor probes. The sensor response is then analysed using machine learning tools to predict the *S. aureus* strain.

The underlying principle of the current work is to exploit the cross‐reactivity of aptamers (specifically created to detect *S. aureus*) to different strains of *S. aureus*. We envisaged that exposing a suite of aptamer‐GNP conjugates to different strains of *S. aureus* would result in a unique colorimetric fingerprint for each strain. This colorimetric fingerprint can then be analysed using machine‐learning algorithms [50, 51, 52, 53, 54] using a train‐and‐test approach to enable the accurate identification of *S. aureus* strains (Scheme 3). This approach is inspired by the way our sensory system detects subtle flavour profiles in food and wine through a *pattern identification–recognition* process [37, 55].

To achieve this, a panel of four aptamers that bind to unique sites on the surface of *S. aureus* was carefully chosen (*viz*., *SA20*, *SA23*, *SA31*, and *SA43*), as shown in Scheme 1 (aptamer sequences presented in Table S2) [4]. Each aptamer was then independently exposed to enzymatically active GNPs as a function of aptamer concentration. As the aptamer concentration increased, a consistent decrease in the enzymatic activity of GNPs was observed in all cases (Figure S5). This observation is consistent with our previous studies, in which the structural flexibility of aptamers allowed them to passivate the GNP surface *via* electrostatic interactions [44, 45, 46, 47]. A nonlinear least‐squares fitting revealed a distinct dissociation constant (*k_d_*) and Hill coefficient (association stoichiometry) for each aptamer. A Hill coefficient value >1 was observed in all cases, suggesting a positive cooperative binding behaviour [47]. The dissociation constant in all cases was in the nM range, with the lowest observed for *SA43* and the highest for *SA31*. We recently showed that such interactions play a critical role in the dissociation of the aptamer from the surface of the GNPs when exposed to the aptamer‐specific target [45, 47]. Stable measurements where a maximum loss of nanozyme activity was observed were chosen as the appropriate aptamer concentration for fabricating the sensor probe (200 nm). The number of aptamers bound to the surface of each GNP nanozyme was calculated to be approximately 250, suggesting multiple aptamer layers are present on the surface of GNPs (calculation is detailed in Supporting Information). Particular care was taken to ensure that the GNPs remained stable during sensor probe fabrication (see Figure S6 for GNP and GNP–aptamer stability).

**FIGURE 1:**
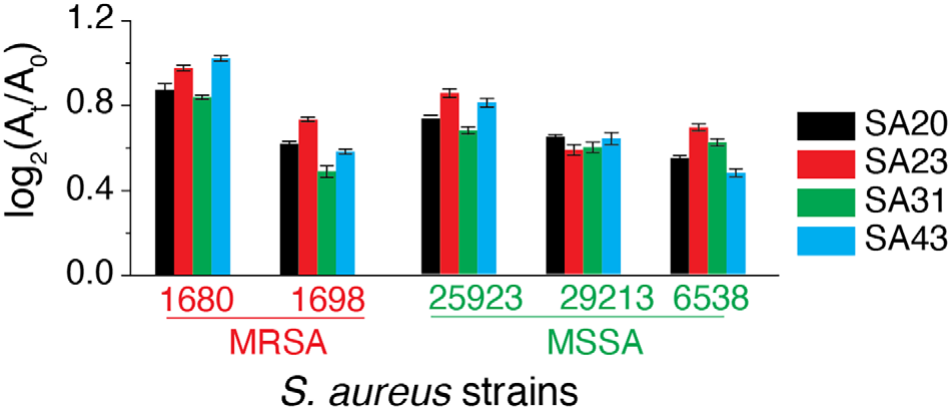
Sensor response following incubation of the sensor probes with five different *S. aureus* strains. A_t_ and A_0_ are the absorbance readings before and after the addition of the peroxidase substrate, TMB and H_2_O_2_, respectively. The data shown are the average of eight independent replicates, and the error bars represent ± SD.

As the dissociation of the aptamer from the surface of GNPs is central to the sensor mechanism, we first assessed whether the sensor probes regained their enzymatic catalytic activity in the presence of the target pathogen, *S. aureus*. Each aptamer binds to different surface sites on *S. aureus* [4] and shows a distinct binding affinity to GNPs. Therefore, we anticipated that exposure to a fixed number of *S. aureus* cells would generate a unique colorimetric response for each probe. Consistent with this anticipation, when the probes were independently exposed to different strains of *S. aureus* (10^5^ cells), a unique colorimetric response was observed depending on the affinity of each aptamer to the strain (Figure 1).

In addition to *S. aureus*, we exposed the probe to several pathogens based on the following criteria: (a) common pathogens involved in nosocomial and/or bacterial infections, (b) high pathogenicity, (c) structural differences in the cell wall (Gram‐positive *vs*. Gram‐negative), and (d) overall shape of bacteria. Based on these, five pathogens were chosen as targets, including *Escherichia coli* (Gram‐negative, rod‐shaped, nosocomial), *Listeria monocytogenes* (Gram‐positive, rod‐shaped, non‐nosocomial), *Pseudomonas aeruginosa* (Gram‐negative, rod‐shaped, nosocomial), *Staphylococcus epidermidis* (Gram‐positive, cocci, nosocomial) and *Streptococcus agalactiae* (Gram‐positive, cocci, non‐nosocomial). In the case of other pathogens, a minimal response was observed (Figure S7) compared to the colorimetric response observed for *S. aureus* strains (10^5^ cells used in all cases). Given the minimal sensor response to non‐specific pathogens, we established that the aptamers showed high selectivity for *S. aureus*.

We also evaluated the quantitative sensing performance of the aptasensor platform by exposing the array to increasing *S. aureus* cells ranging from 10^2^ to 10^5^ cells. An increase in the sensor response was observed with increasing bacterial concentration, demonstrating that the platform is responsive across a broad dynamic range (Figure S8). A detectable signal was obtained at concentrations as low as 10^2^ cells, indicating a low analytical limit of detection. To understand the practical limit of detection, the responses from non‐target bacterial species at 10^5^ cells were taken into consideration, with the maximum background signal approximated by a threshold value of ∼0.2. Notably, *S. aureus* responses exceeded this background threshold from 10^3^ cells onward, enabling reliable discrimination from non‐target organisms.

The fingerprint responses from each *S. aureus* strain (10^5^ cells) were used to build a statistical model to discriminate the unique signatures obtained for each *S. aureus* strain. An unsupervised machine learning tool, such as hierarchical cluster analysis (HCA), was first used to classify different *S. aureus* strains into separate branches of a cluster dendrogram. In this work, an Euclidean metric with a “*Furthest neighbour*” method of linkage was used. At first glance, the clustering of the different *S. aureus* strains appeared random, as MRSA 1680 and MSSA 25923 were clustered together within a clade (Figure S9a). Further analysis showed that the clustering was, in fact, a reflection of the presence or absence of the *pvl* (Panton‐Valentine Leukocidin gene) virulence gene. The expression of *pvl* with MRSA presents a challenging conundrum in disease management as it requires improved vigilance and detection [7]. Based on these results, it is evident that the first line of HCA classification is based on the presence or absence of the *pvl* gene (*pvl–* strains 6538, 29213 and 1698 clustered together within a clade and *pvl+* strains 25923 and 1680 clustered together in a different clade), and the second line of classification is based on their sensitivity/resistivity to the antibiotic, methicillin (strains 6538 and 29213 clustered together and MRSA 1698 in a different leaf). Similarly, strains 1680 and 25293 were clustered in a single clade, as both expressed the *pvl* gene, but were clustered in a separate leaf. The responses were also analysed using a supervised machine learning tool, such as linear discriminant analysis (LDA), a statistical model that transforms multivariate data into a reduced number of variables through orthogonal linear combinations [37, 56]. LDA showed five non‐overlapping independent clusters corresponding to the five strains (Figure S9b). The quality of the LDA classifier was confirmed using a leave‐one‐out cross‐validation analysis. Jackknife analysis on the training set (5 strains × 8 replicates × 4 channels) revealed 100% between‐group cross‐validation accuracy, signifying that discriminant analysis is a robust tool for this sensing approach. The LDA used to discern the signatures was used as a training set, and a new set of sensor responses was used to predict the strains. In all cases, the sensor could accurately predict each strain (Figure S9b—pink dots). Taken together, we propose that the absorbance fingerprint resulting in the unique clustering of the different strains was due to surface characteristics that are influenced by virulence factors and sensitivity to antibiotics. The ability to stratify small changes demonstrated the ability of our sensor to detect (‘*smell’*) subtle changes. This would mean that if an unknown strain is exposed to the array‐based aptasensor, it can not only detect *S. aureus* but also predict strain features, such as the presence or absence of virulent genes and susceptibility to antibiotics.

**FIGURE 2:**
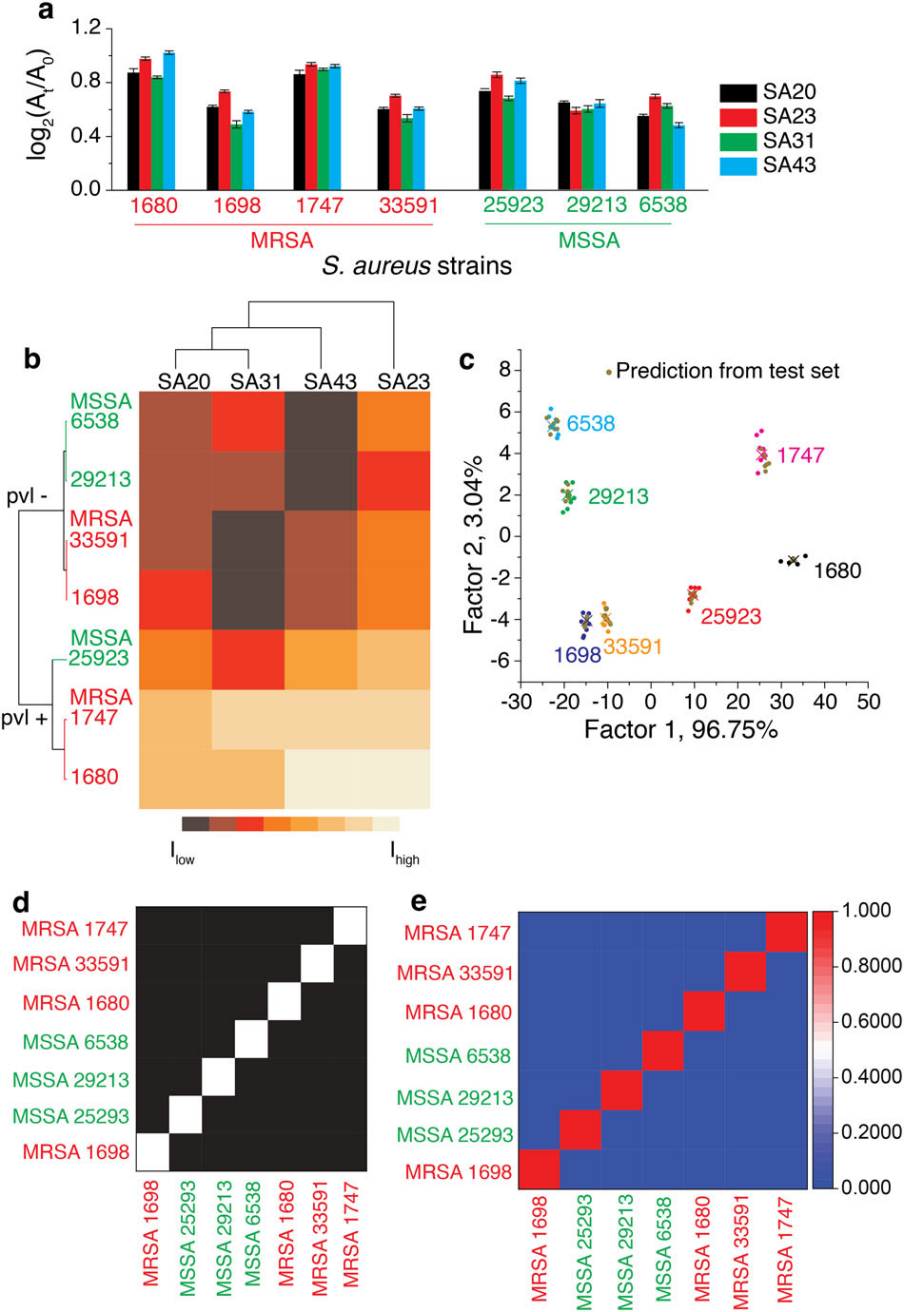
(a) Sensor response following incubation of the sensor probes with seven different *S. aureus* strains in buffer. A_t_ and A_0_ are the respective absorbance readings before and after adding the peroxidase substrate, TMB and H_2_O_2_. The data shown are the average of eight independent replicates, and the error bars represent ± SD, (b) the heat map and cluster dendrogram generated using an unsupervised machine learning tool, HCA, (c) supervised machine learning tool, LDA of the absorbance signatures resulting in canonical scores with two discriminants explaining 96.75% and 3.04% of the total variance, (d) Leave‐one‐out cross‐validation showing 100% correct classification, where each sample was accurately identified when excluded from the training set, and (e) Confusion matrix of the Naïve Bayes classifier showing class‐wise prediction accuracy, with only minor misassignments observed between the most closely related classes.

Having established the ability of the sensor to classify different *S. aureus* strains, we validated whether the clustering was accurate in predicting (or ‘*smelling*’) subtle differences on the surface of *S. aureus*. For this purpose, we introduced two additional *S. aureus* strains, both of which were resistant to methicillin. However, one strain expressed the *pvl* gene (1747), while the other was a *pvl–* strain (33591). A colorimetric fingerprint unique to each strain was first obtained (Figure 2a). HCA analysis using Euclidean metric with a “*Furthest neighbour*” method of linkage showed two broad clades based on the presence or absence of the *pvl* gene. Each clade was further subdivided based on its sensitivity/resistance to methicillin. In this case, the HCA dendrogram clustered strains 1680 and 1747 in the *pvl*+ MRSA clade, whereas strains 1698 and 33591 clustered in the *pvl–* MRSA clade (Figure 2b). LDA revealed seven independent clusters corresponding to the seven S. aureus strains (Figure 2c). While each strain could be accurately identified using the test set, partial overlap or proximity between certain clusters (e.g., strains 1698 and 33591, 1680 and 1747) suggests that these strains share more similar feature profiles compared to others. Furthermore, the overlap of the unknowns with individual clusters in the LDA plot showed 100% accuracy in predicting these *S. aureus* strains. This demonstrates that the combination of an array‐based sensor system with supervised/unsupervised machine learning tools provides significant advantages in detecting *S. aureus* at the strain level.

The robustness of the classification model was evaluated using multiple complementary validation strategies, including leave‐one‐out cross‐validation and extensive bootstrapping. The jackknife estimation showed 100% accuracy, indicating that the classifier maintained consistent predictive performance even when each sample was systematically removed from the training set (Figure 2d). This result suggests that the model is not overly dependent on any single data point and that the underlying feature set offers strong discriminatory power across all classes. To further quantify classification performance, a Naïve Bayes confusion matrix created using 1000 bootstrap iterations was generated, which showed an overall correct classification rate (CCR) of 99.5% (Figure 2e). The minimal misclassification observed (<1%) indicates highly reliable predictive capability, even when accounting for intrinsic variability among the samples. The narrow distribution of CCR values across bootstrap replicates demonstrates excellent reproducibility, confirming that the high accuracy is not an artefact of the sample composition but reflects a robust underlying pattern recognisable by the Naïve Bayes classifier. Together, these findings highlight that the model is both accurate and robust, with evidence from leave‐one‐out cross‐validation and confusion‐matrix analysis. This multi‐layered validation framework underscores the reliability of the classification approach and supports its use for predictive sensing of *S. aureus* strains.

Randomisation of each *S. aureus* strain into a discovery/training set, followed by comparison of each strain against other strains in the test set using Akaike information criterion (AIC) analysis, enabled the identification of the key aptamer for each strain (Figure S10). The key aptamers were further validated using receiver operating characteristic (ROC) curve analysis. Based on this analysis, aptamer *SA43* was a common key predictor for five *S. aureus* strains, whereas aptamers *SA31*, *SA23* and *SA20* were common predictors in four, three, and one of the seven *S. aureus* strains, respectively (Table 1). These observations suggest that, similar to human olfaction [37, 55], the current sensor can be “*trained*” to recognise *S. aureus* strains using appropriate algorithms. This trainability makes such sensors highly versatile, enabling predictable detection of new *S. aureus* strains without the need to redesign the sensor array.

**TABLE 1:** ROC analysis of the AIC‐selected aptamers from the discovery set applied to the test set of *S. aureus* strain samples (aptamer 1 = *SA20*, aptamer 2 = *SA23*, aptamer 3 = *SA31* and aptamer 4 = *SA43*).

	Validation/test set ROC analysis						
AIC model	Specificity	Sensitivity	True negative	True positive	False negative	False positive	AUC
Apt 4	1	1	8	48	0	0	1
Apt 2+3	1	1	8	48	0	0	1
Apt 2+3+4	1	1	8	48	0	0	1
Apt 1+3+4	1	1	8	48	0	0	1
Apt 2+4	1	1	8	48	0	0	1
Apt 3+4	0.88	1	7	48	0	1	0.96
Apt 2	1	1	8	48	0	0	1

**FIGURE 3:**
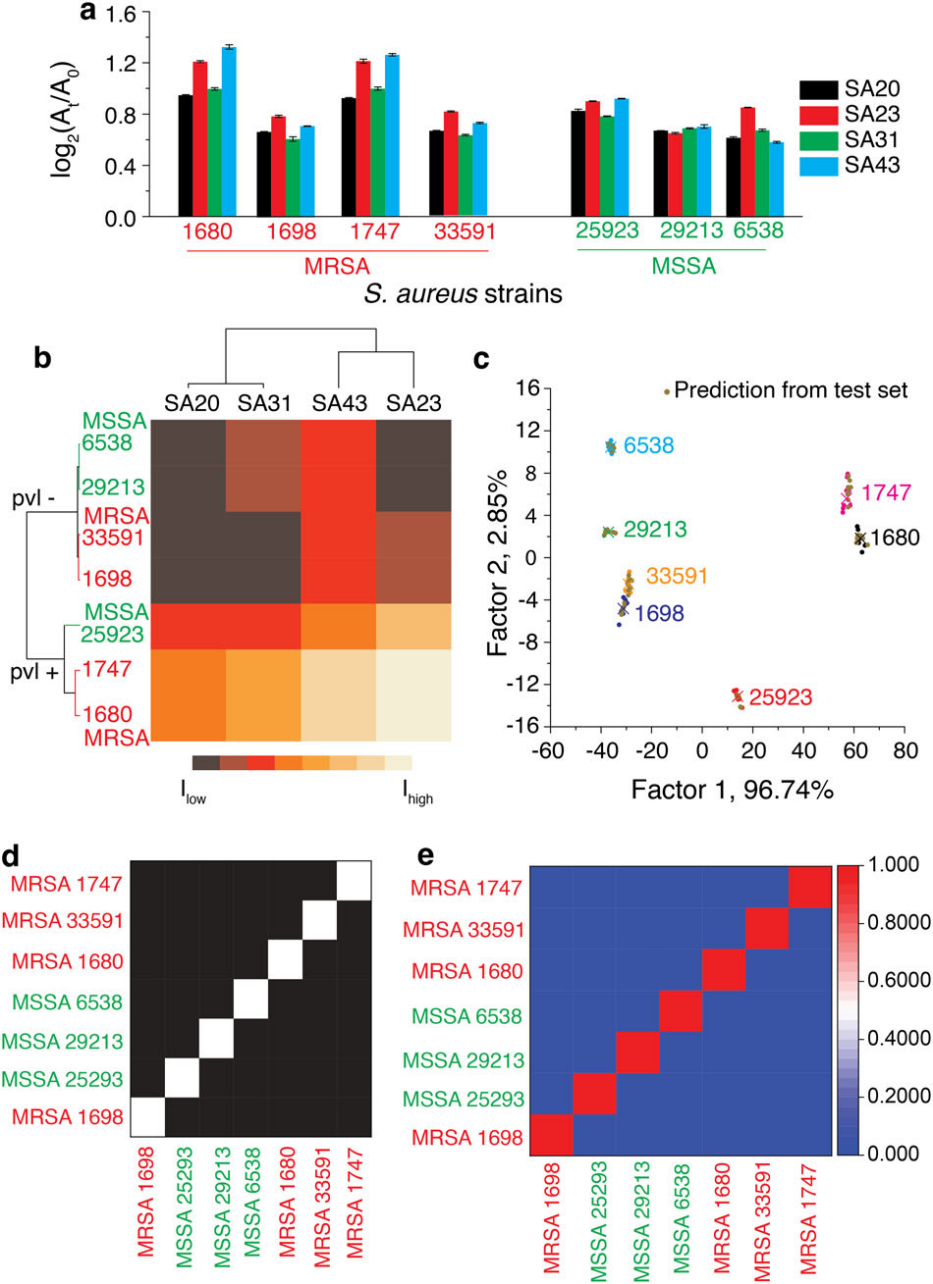
(a) Sensor response following incubation of the sensor probes with seven different *S. aureus* strains in the SWF. A_t_ and A_0_ are the respective absorbance readings before and after adding the peroxidase substrate, TMB and H_2_O_2_. The data are an average of eight independent replicates, and the error bars represent ± SD, (b) the heat map and cluster dendrogram generated using HCA, (c) LDA of the absorbance signatures resulting in canonical scores with two discriminants explaining 96.74% and 2.85% of the total variance, (d) Leave‐one‐out cross‐validation showing 100% correct classification, where each sample was accurately identified when excluded from the training set, and (e) Confusion matrix of the Naïve Bayes classifier showing class‐wise prediction accuracy, with only minor misassignments observed between the most closely related classes.

We then validated the sensor performance using a simulated wound fluid (SWF). The colorimetric fingerprint response for each strain (Figure 3a) was similar to that observed for the buffer (Figure 2a), with one notable exception. The sensor response in the SWF was more intense and rapid than that observed under the buffer conditions. This is possibly due to the presence of salts (in SWF) that can facilitate aptamer folding when they desorb from the surface of GNPs, an observation that we have seen previously [12, 39, 40]. To understand the origin of this enhanced response, we conducted complementary structural prediction and thermodynamic analyses, the full details of which are provided in the Supporting Information. Briefly, secondary structure prediction (mFold) under ionic conditions representative of buffer and SWF indicated no significant changes in aptamer folding (Figure S11). In contrast, thermodynamic analysis revealed a consistent stabilisation of aptamer folding in SWF, reflected by more favourable Gibbs free energies and increased melting temperatures (Table S3). Together, these results suggest that SWF promotes a greater fraction of aptamers to remain in the correct, binding‐competent conformation at the assay temperature and thereby improves sensor sensitivity and response kinetics. The use of unsupervised HCA showed similar clustering, where the first line of classification was based on the presence or absence of the *pvl* gene, and the second line of classification was based on the sensitivity/resistance of the strain to the antibiotic, methicillin (Figure 3b).

A key change in the HCA dendrogram was the clustering of the aptamers. For instance, in buffer conditions, aptamers *SA20* and *SA31* clustered in a single clade, whereas *SA43* and *SA23* were independent. Conversely, in SWF, *SA43* and *SA23* were clustered together, indicating similar responses under these conditions. This change suggests that the change in the composition of buffer *vs*. SWF influences aptamer binding affinities and/or dissociation rates. LDA clustering also showed seven non‐overlapping independent clusters (Figure 3c), similar to those observed in Figure 2c, and showed 100% accuracy in predicting these *S. aureus* strains. The robustness of the classification model evaluated using leave‐one‐out cross‐validation showed 100% correct classification, where each sample was accurately identified when excluded from the training set. The confusion matrix of the Naïve Bayes classifier achieved an overall correct classification rate of 99.2%, with only minor misassignments observed between the most closely related classes.

In summary, we have, for the first time, developed an array‐based nanozyme aptasensing platform for strain‐level detection of *S. aureus* by incorporating multiple molecular recognition elements (aptamers). The unique colorimetric fingerprint obtained for each strain allowed the sensor to readily discriminate phenotypic changes in *S. aureus* based on subtle differences in the cell surface without prior knowledge of the specific antigens on the bacterial surface. The ability to obtain vital information about the strain, sensitivity/resistivity to antibiotics, and the presence or absence of virulence genes will help in informed decision‐making for administering appropriate treatment options. Furthermore, the ability of the sensor to detect subtle changes provides a unique opportunity to predict new antibiotic‐resistant strains without the need to fully redevelop the sensor. Importantly, to contextualise the translational relevance of this work, we compared the proposed platform with commonly used diagnostic approaches, including PCR, culture‐based assays, and ELISA (Table S4). While PCR and culture methods remain the gold standard for definitive clinical confirmation, they require specialised laboratory infrastructure, trained personnel, and extended turnaround times. In contrast, the presented platform is best positioned as a rapid, low‐cost, screening and triage tool, capable of strain‐level discrimination without prior sequence information, predicting virulence‐associated markers, and recognising previously unencountered strains through pattern‐recognition‐based fingerprinting. Beyond *S. aureus*, this array‐based strategy provides a versatile framework that can be extended to other clinically relevant pathogens and further enhanced by increasing sensor dimensionality through the incorporation of additional target‐specific aptamers.

## Author Contributions

The manuscript was written through the contributions of all authors. All authors have given approval to the final version of the manuscript.

## Conflicts of Interest

The authors declare no conflicts of interest.

## Supporting information




**Supporting File**: smll72388‐sup‐0001‐SuppMat.docx.

## Data Availability

The data that support the findings of this study are available from the corresponding author upon reasonable request.
