# Supplementary material for: Nanozyme Aptasensor Array for Predictive Sensing of Virulent and Antibiotic‐Resistant Staphylococcus Aureus strains
Source: Small. 2026 Feb 11;22(15):e12266. doi: 10.1002/smll.202512266 (PMC12980457; doi:10.1002/smll.202512266)
Supplement: Supplementary file 1 — Supporting File: smll72388‐sup‐0001‐SuppMat.docx. [file SMLL-22-e12266-s001.docx]

**Supporting Information**

**for**

**Nanozyme Aptasensor Array for Predictive Sensing of Virulent and Antibiotic-Resistant Staphylococcus aureus strains**

Pabudi Weerathunge,^a^ Mahdieh Yazdani,^b^ Tarun K. Sharma,^c^ Wilson K. M. Wong,^d^ Mugdha V. Joglekar,^d^ Anandwardhan A. Hardikar,^d^ Vincent M. Rotello,^b^ Rajesh Ramanathan^a^* and Vipul Bansal^a^*

^a^ Sir Ian Potter NanoBioSensing Facility, NanoBiotechnology Research Laboratory (NBRL), School of Science, RMIT University, GPO Box 2476, Melbourne, VIC 3000, Australia.

^b^ Department of Chemistry, University of Massachusetts Amherst, 710 North Pleasant Street, Amherst, Massachusetts 01003, United States.

^c^ Department of Medical Devices, National Institute of Pharmaceutical Education and Research, Mohali, Punjab 160062, India

^d^ Diabetes and Islet Biology Group, School of Medicine, Western Sydney University, Campbelltown, NSW, Australia.

Materials and reagents

Gold (III) chloride (HAuCl_4_.3H_2_O) and trisodium citrate (Na_3_C_6_H_5_O_7_) were purchased from Sigma-Aldrich (St. Louis, USA). 3,3,5,5-tetramethylbenzidine (TMB) substrate reagent kit and 30% w/w hydrogen peroxide (H_2_O_2_) were purchased from BD Sciences and Chem Supply, respectively. All aptamers were custom-synthesised and obtained from Integrated DNA Technologies Inc. (IDT, USA). All bacterial cultures were grown in appropriate broths purchased from Amyl Media Pty Ltd. Deionised MilliQ water (18.2 MΩ cm) was used in all the experiments and acquired from the Millipore water purification system.

Synthesis of citrate-capped gold nanoparticles.

The citrate-functionalised gold nanoparticles (GNPs) were synthesised using the Turkevich citrate reduction method (**Figure S1**).^1^


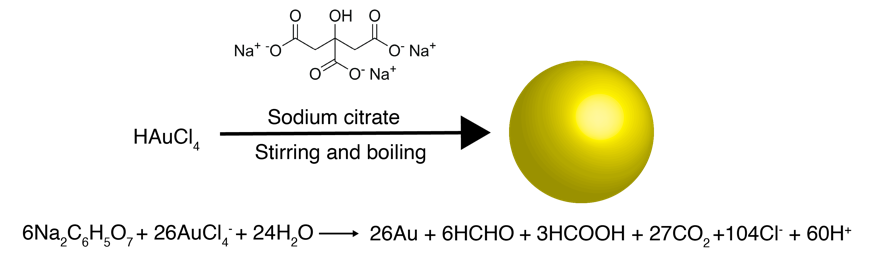


**Figure S1**. Schematic representation of the GNP synthesis.

To synthesise GNPs, 100 mL of 1 mM HAuCl_4_ was added to a flat bottom flask. Under vigorous stirring and boiling, 10 mL of 38.8 mM sodium citrate was injected into the flask, and the solution was stirred for 30 minutes. The colour of the solution changed from pale yellow to wine red. The solution was allowed to cool to room temperature. The unreacted citrate and gold ions were removed by dialysis over 24 h using 12 kDa molecular cut-off cellulose dialysis membranes (water was changed two times). The concentration of the dialysed GNPs was determined by atomic emission spectroscopy using a Varian Fast Sequential AAS after digesting GNPs in aqua regia.

Characterisation of the citrate-capped gold nanoparticles.

The GNPs were characterised using a suite of material characterisation techniques, including microscopy (transmission electron microscopy - TEM), spectroscopy (UV-visible spectroscopy, X-ray diffraction spectroscopy - XRD), dynamic light scattering (DLS), and zeta potential measurements.


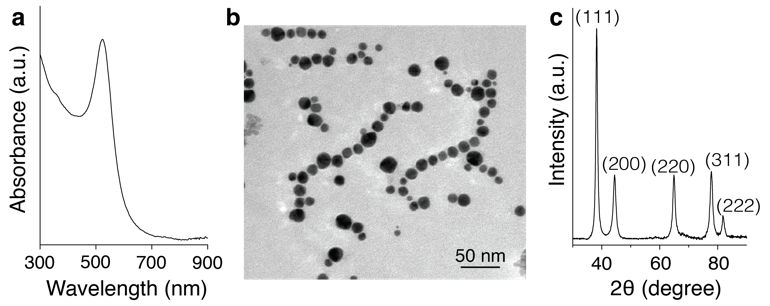


**Figure S2**. Characterisation of GNPs. (a) UV-visible spectrum showing the characteristic SPR peak at 530 nm; (b) TEM image confirming the quasi-spherical morphology of the GNPs; and (c) XRD spectrum showing typical Bragg reflections corresponding to the fcc gold planes.

The UV-visible absorption spectrum obtained using a Varian Cary 50 spectrophotometer showed a sharp characteristic surface plasmon resonance band (SPR) at 530 nm, with no additional bands in the near-infrared region (Figure S2a). This SPR feature is typical of quasi-spherical GNPs, whereas the peak sharpness suggests a narrow size distribution range.^2-4^ The morphology was confirmed using a JEOL 1010 TEM instrument operated at an accelerating voltage of 100 kV, in which the GNPs were drop-coated onto a carbon-coated Cu TEM grid. The TEM image confirmed that the GNPs were quasi-spherical (**Figure S2**b) with an average diameter of *ca*. 15 ± 1.1 nm. XRD was performed using a Bruker AXS D4 Endeavour wide-angle X-ray diffractometer with Cu Kα radiation (λ = 1.5406 Å). The GNPs exhibited well-defined Bragg reflections corresponding to the (111), (200), (220), (311), and (222) Miller indices, which could be indexed to the face-centred cubic lattice structure of crystalline Au (Figure S2c).

Evaluation of enzyme-mimic catalytic activity (nanozyme activity) of citrate-capped GNPs.

The nanozyme activity was evaluated by assessing the ability of GNPs to oxidise chromogenic substrate 3,3',5,5'-Tetramethylbenzidine (TMB) to form a blue-coloured product in the presence of H_2_O_2_ (blue colour measured using a Perkin Elmer multimode plate reader). The oxidation of TMB leads to a relatively stable charge transfer complex, characterised by a typical absorbance at 652 nm (**Figure S3**a).^5^


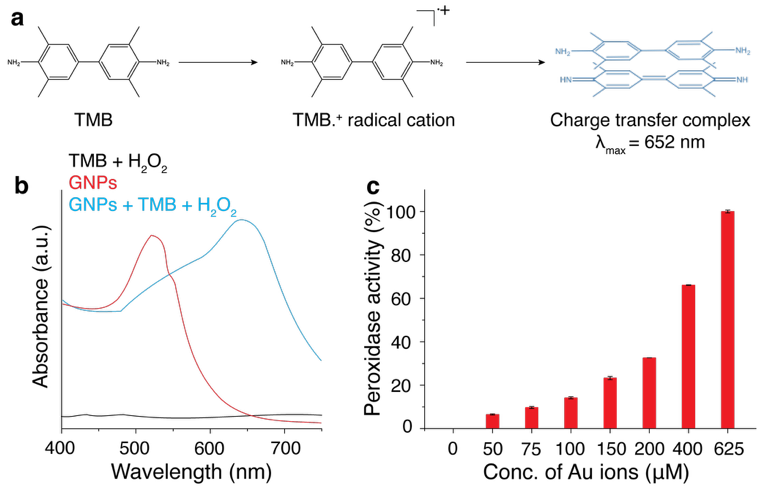


**Figure S3**. The peroxidase-mimic catalytic activity of GNPs. (a) Schematic illustration of the pathway followed during the oxidation of chromogenic substrate TMB; (b) UV-visible spectrum showing the oxidation of TMB facilitated by GNPs in the presence of H_2_O_2_ (𝛌_max_ = 652 nm); (c) influence of Au ion concentration in GNPs on the peroxidase-mimic catalytic activity.

The citrate-capped GNPs oxidised TMB in the presence of H_2_O_2_ as the co-substrate (Figure S3b). The catalytic activity improved with increasing concentration of GNPs (Figure S3c), an observation akin to other nanozymes.^3, 4, 6-9^


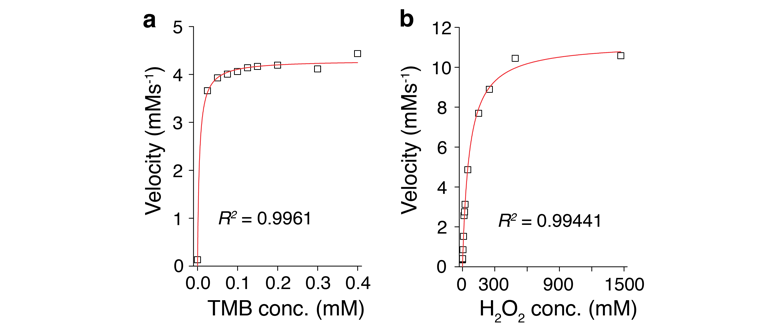


**Figure S4**. Calculation of enzyme kinetic parameters. Increase in reaction velocity as a function of increasing (a) TMB and (b) H_2_O_2_ concentrations.

Similar to other nanozymes, the peroxidase-mimicking activity of citrate-capped GNPs was dependent on TMB concentration (Figure S4a) and H_2_O_2_ concentration (Figure S4b). Enzyme kinetic theory was then used to determine kinetic parameters, such as Michaelis constant – *K_m_*, maximum initial velocity – *V_max_* and turnover number – *K_cat_*. The reaction velocity for increasing H_2_O_2_ and TMB concentrations was fitted using a non-linear curve fit (independently) that showed characteristic Michaelis-Menten curves (Figure S4). All fittings were performed using OriginPro 2016 with the Michaelis-Menten enzyme kinetic model and Leveneberg-Marquardt iteration algorithm. The *K_m_* and *V_max_* were then obtained, where the *K_m_* represents the affinity of the nanoparticle to the substrate (lower *K_m_* value = higher affinity). The catalytic constant, *K_cat_*, also known as the turnover number, was calculated using the following equation:

*K_cat_* = *V_max_* / [E]

where [E] is the concentration of nanoparticles. The obtained values are comparable to those reported in the literature for GNPs.

**Table S1**. Enzyme kinetic parameters for citrate-capped GNPs.

| **Substrate** | ***K_m_* (mM)** | ***V_max_* (mM/s)** | ***K_cat_* (/s)** |
| --- | --- | --- | --- |
| TMB | 0.005 | 4.3 × 10^-5^ | 5.7 × 10^-4^ |
| H_2_O_2_ | 62.7 | 1.1 × 10^-4^ | 1.5 × 10^-3^ |

Based on these results, the optimum parameters for sensor probe fabrication were determined as follows: GNP concentration = 75 µM, temperature = 37 °C (because it does not affect the bacteria), TMB concentration = 200 µM, and H_2_O_2_ concentration = 1 mM.

Fabrication of aptamer-GNP conjugates.

The sensor probes were fabricated by exposing four aptamers (details of aptamers in Table S2) to the GNPs independently.

**Table S2.** Details of aptamer sequences used to fabricate sensor probes. The aptamers were chosen from ref# 10.

| Aptamer name | Aptamer sequence (5’-3’) |
| --- | --- |
| *SA20* | GCGCCCTCTCACGTGGCACTCAGAGTGCCGGAAGTTCTGCGTTAT |
| *SA23* | GGGCTGGCCAGATCAGACCCCGGATGATCATCCTTGTGAGAACCA |
| *SA31* | TCCCACGATCTCATTAGTCTGTGGATAAGCGTGGGACGTCTATGA |
| *SA43* | TCGGCACGTTCTCAGTAGCGCTCGCTGGTCATCCCACAGCTACGT |

*all aptamers were custom-synthesised through Integrated DNA Technologies (IDT, USA)

The aptamers were first heated (92 °C) for 10 min, followed by snap-chilling on ice for 5 min and allowed to cool to room temperature. Different concentrations (0-300 nM) of the aptamers were incubated with a fixed concentration of GNPs (75 µM) for 10 min at 37 °C. The nanozyme activity of the GNP-aptamer conjugate was assessed by adding TMB/H_2_O_2_ and monitoring the oxidation product of TMB at 652 nm.


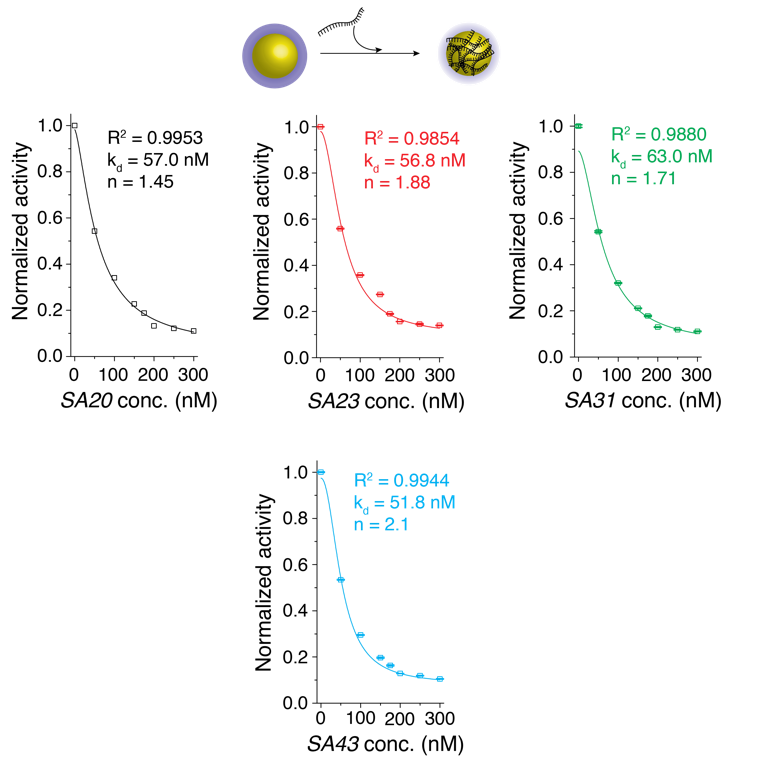


**Figure S5**. Inhibition of the catalytic activity of GNPs. The inhibition of the catalytic activity of GNPs as a function of increasing concentrations of (a) *SA20*, (b) *SA23*, (c) *SA31*, and (d) *SA43* aptamers.

As shown in Figure S5, the nanozyme activity of GNPs decreased with increasing aptamer concentration because the structural flexibility of aptamers facilitated electrostatic interactions, allowing them to passivate the GNP surface. Previous studies have suggested that the structural flexibility of ssDNA allows it to uncoil and expose its nitrogenous bases to GNPs, leading to a coordination interaction between the nitrogenous bases and GNPs.^11^ It is also evident that the nanozyme activity is reduced by ~90% when using a 200 nM concentration of aptamers. Therefore, this concentration was chosen for fabricating the sensor probes used for further sensing studies. The non-linear least squares fitting of the aptamer-GNP interactions revealed the dissociation constant and Hill coefficient (association stoichiometry). As shown in Figure S5, an average *K_d_* of 57.2 nM and a Hill coefficient value of > 1 were obtained. A Hill coefficient value of >1 reflects a positive cooperative binding behaviour, such that binding of one aptamer to the GNP enhances the affinity of another aptamer to bind to its surface. The observed *K_d_* values for the different aptamer-GNP interactions corroborate well with the previously observed values for stable, non-covalently bonded biomolecule-GNP systems.ref

We also estimated the number of aptamers required to block a single GNP’s nanozyme activity. For this purpose, we first calculated the number of GNPs in the reaction using the mass of each NP with an average diameter of ~15 nm. Considering that the average diameter of the GNPs was 15 nm, the volume of each GNP was 1.8 × 10^-18^ cm^3^. As the density of Au is 19.32 g/cm^3^, the mass of each GNP is 3.4 × 10^-17^ g. The number of moles of Au in the reaction is 1.5 × 10^-8^ moles, which corresponds to 3.0 × 10^-6^ g of Au (the molecular weight of Au is 196.96657). Therefore, the number of GNPs in the reaction corresponds to 8.7 × 10^10^ particles. The number of aptamers can be calculated based on the moles of aptamers. For the fabrication of the sensor probe, 200 nM aptamers were used (100 µL volume), and the total moles of aptamers available to interact with the GNPs correspond to 4.0 × 10^-11^ moles, which is equivalent to 2.4 × 10^13^ aptamer molecules. Therefore, each GNP can potentially interact with an average of ~278 aptamers (considering 100% binding).

The potential aptamers that interact with GNPs can be calculated by considering the surface area of the aptamers and the nanozyme. In this instance, both the size of the aptamer and the nanozyme must be considered. Each aptamer used in this study contained 45 base pairs. Considering a distance of 0.676 nm between the two base pairs and 0.33 nm width of the DNA, the total surface area of each aptamer would be 5.0 nm^2^. Similarly, considering an average diameter of 15 nm for the GNPs, the surface area of each nanoparticle was 706.8 nm^2^. Therefore, each GNP could be blocked by an average of ~140 aptamers.

Based on Figure S5, ~90% quenching of the nanozyme activity was achieved during the formation of the sensor probe. If this is considered for the calculation, the number of aptamers interacting with the GNPs would be ~ 250. This suggests that multiple layers of aptamers were formed during the formation of the sensor probe, as is also evident from the Hill coefficient, which suggests that the binding of one aptamer to the GNP facilitates the binding of additional aptamers.


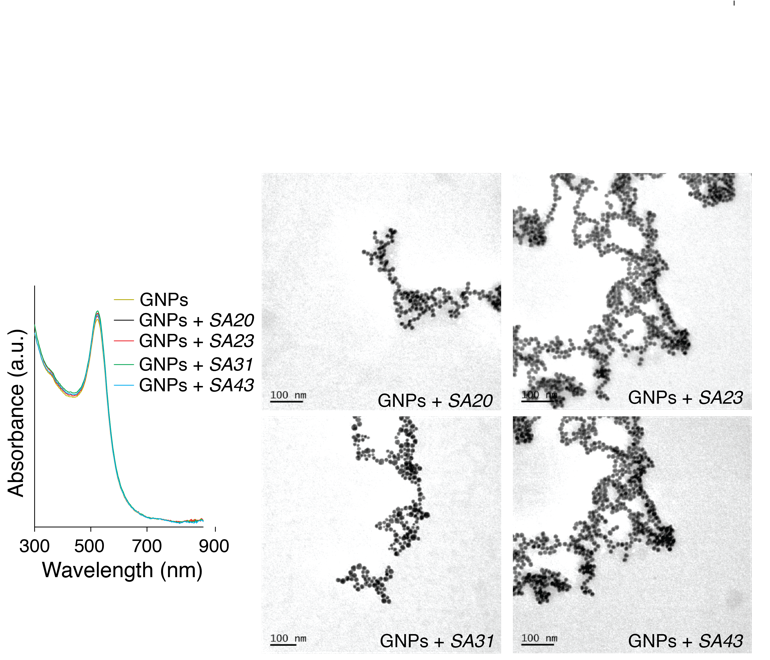


**Figure S6**. Stability of GNP-aptamer conjugates. UV-visible spectra and corresponding TEM images of GNPs + *SA20*, GNPs + *SA23*, GNPs + *SA31*, and GNPs + *SA43* obtained at the highest aptamer concentration used for the generation of the final sensor probe (200 nM).

We also assessed the stability of the GNP-aptamer conjugates by UV-visible spectroscopy and TEM. The UV-visible spectra obtained from the GNP-aptamer conjugates showed an SPR band at ca. 530 nm (Figure S6), similar to that obtained for pristine GNPs (Figure S2a). This suggests that the GNP-aptamer conjugates were stable, even after the formation of the sensor probe. TEM analysis further validated the stability of the GNPs following passivation with aptamers, wherein no change in the size of the NPs was observed.

Preparation of bacterial cultures.

ATCC strains of *Staphylococcus aureus* (1680, 1698, 1747, 33591, 25923, 29213, and 6538), *Staphylococcus epidermidis* ATCC 12228, *Streptococcus agalactiae*, *Listeria monocytogenes*, *Escherichia coli* and *Pseudomonas aeruginosa* were procured from the RMIT Microbiology Department and were maintained and cultured in nutrient broth (NB). The bacteria were grown, and the optical density at 600 nm was maintained at 1.0. The cells were collected by centrifugation (performed at 4 °C) at 5000 rpm for 10 minutes, and the pellets were washed three times with 10x phosphate buffer saline (PBS). The cells were fixed with methanol and stored at –20 °C. The fixed bacterial strains were washed three times with 10x PBS and finally dissolved in 1x PBS. The number of bacteria was quantified using a hemocytometer before being used for sensing experiments.

Sensing performance of array-based aptasensor.

The sensor array was exposed to several pathogens (10^5^ cells). These were chosen based on the following criteria: (a) common pathogens involved in nosocomial and/or bacterial infections; (b) high pathogenicity; (c) structural differences in the cell wall (Gram-positive *vs*. Gram-negative); and (d) overall shape of the bacteria. Based on these, five pathogens were chosen as targets, including *Escherichia coli* (Gram-negative, rod-shaped, nosocomial), *Listeria monocytogenes* (Gram-positive, rod-shaped, non-nosocomial), *Pseudomonas aeruginosa* (Gram-negative, rod-shaped, nosocomial), *Staphylococcus epidermidis* (Gram-positive, cocci, nosocomial) and *Streptococcus agalactiae* (Gram-positive, cocci, non-nosocomial).


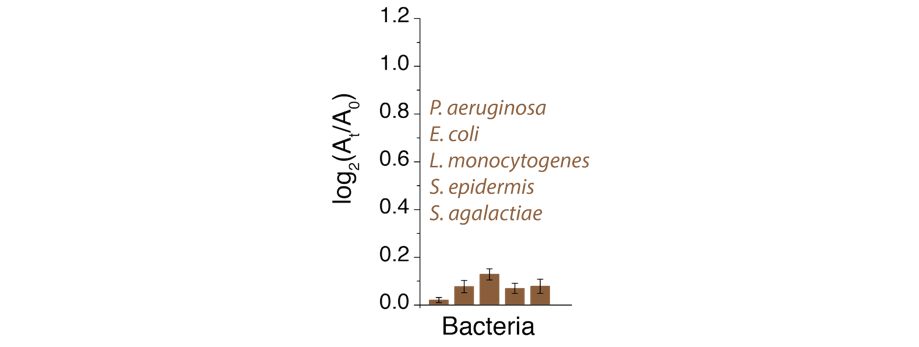


**Figure S7**. Sensor response [absorbance] following incubation of the sensor probes with five different pathogens. A_t_ and A_0_ are the absorbance readings before and after adding the peroxidase substrate, TMB and H_2_O_2,_ respectively. The data shown is an average of eight independent replicates, and the error bars represent ± SD.

As shown in Figure S7, a minimal response was observed in comparison with the colorimetric response observed for the *S. aureus* strains (Figure 1, main manuscript). This indicates that the aptamers were highly specific for binding to *S. aureus*.

To evaluate the quantitative sensing performance of the aptasensor platform, a dose–response study was conducted using increasing concentrations of *S. aureus* cells ranging from 10² to 10⁵ cells (**Figure S8**). An increase in sensor response was observed with increasing bacterial concentration, demonstrating that the platform is responsive across a broad dynamic range. A detectable signal was observed at concentrations as low as 10² cells, indicating a low analytical limit of detection. However, as array-based sensors rely on differential response patterns rather than absolute signal intensity, it is essential to define detection limits in the context of non-specific background responses. To this end, responses from non-target bacterial species at 10⁵ cells were analysed, with the maximum background signal approximated by a threshold value of ~0.2.

Notably, *S. aureus* responses exceeded this background threshold from 10³ cells onward, enabling reliable discrimination from non-target organisms. Consequently, while the analytical LOD is 10² cells, the practical detection limit for selective *S. aureus* identification is conservatively defined as 10³ cells.


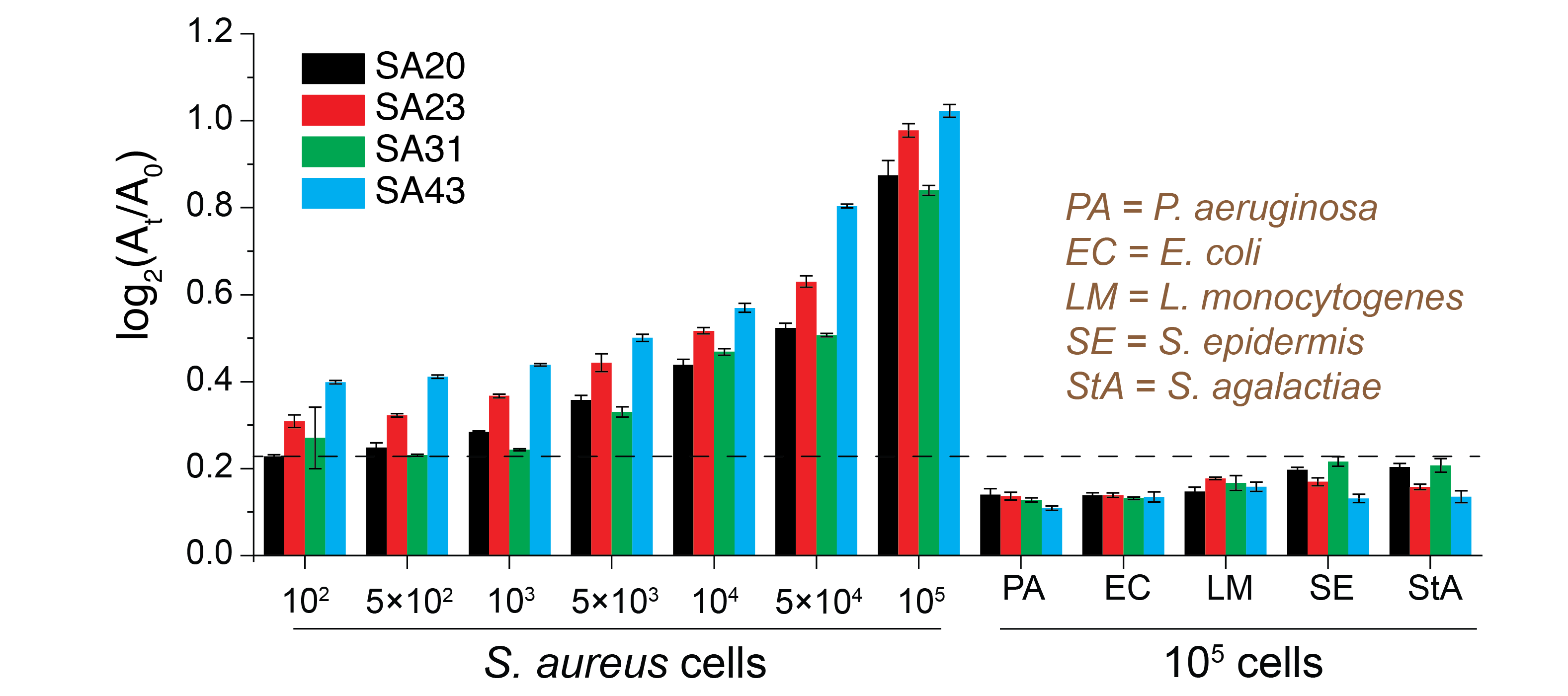


**Figure S8**. Sensor response following incubation of the sensor probes with increasing *S. aureus* cells and five different pathogens. A_t_ and A_0_ are the absorbance readings before and after adding the peroxidase substrate, TMB and H_2_O_2,_ respectively. The data shown is an average of eight independent replicates, and the error bars represent ± SD.

The colorimetric fingerprint obtained from each *S. aureus* strain was used to build a statistical model to discriminate these signatures. An unsupervised machine learning model, such as hierarchical cluster analysis (HCA), was first used to classify different *S. aureus* strains into separate branches of a cluster dendrogram. In this work, an Euclidean metric with a “*Furthest neighbour*” method of linkage was used. At first glance, the clustering of the different *S. aureus* strains appeared random, as MRSA 1680 and MSSA 25923 clustered together within a clade (**Figure S9a**). Further analysis showed that the clustering was, in fact, a reflection of the presence or absence of the *pvl* (Panton-Valentine Leukocidin gene) virulence gene. The expression of *pvl* with MRSA presents a challenging conundrum in disease management as it requires improved vigilance and detection.^12^ Based on these results, it is evident that the first line of classification is based on the presence or absence of the *pvl* gene (*pvl–* strains 6538, 29213 and 1698 clustered together within a clade and *pvl+* strains 25923 and 1680 clustered together in a different clade), and the second line of classification is based on their sensitivity/resistivity to the antibiotic, methicillin (strains 6538 and 29213 clustered together and MRSA 1698 in a different leaf). Similarly, strains 1680 and 25293 were clustered in a single clade, as both expressed the *pvl* gene, but were clustered in a separate leaf.


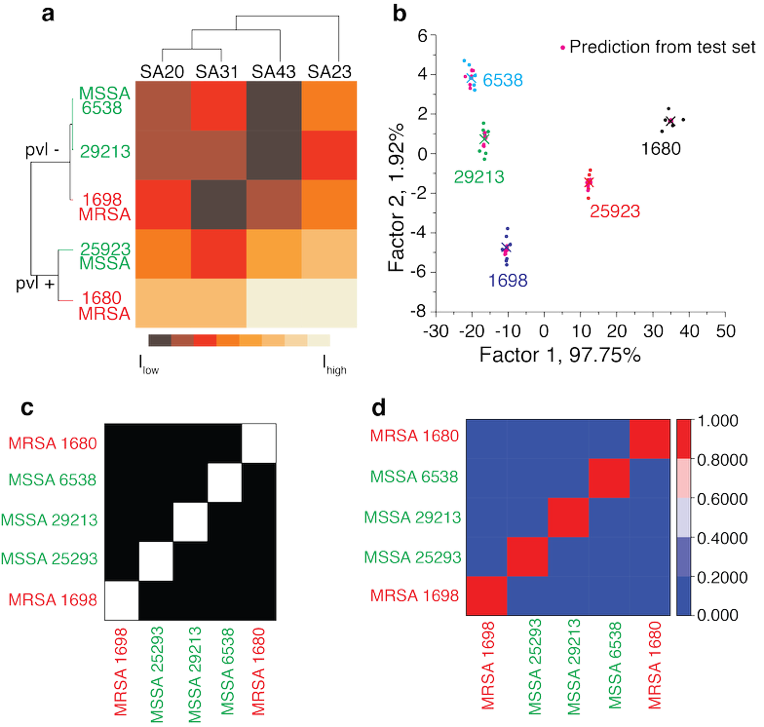


**Figure S9**. (a) The heat map and cluster dendrogram using an unsupervised machine learning tool, hierarchical cluster analysis (HCA); (b) supervised machine learning tool, linear discriminant analysis (LDA) of the absorbance signatures, resulting in canonical scores with two discriminants explaining 97.75% and 1.92% of the total variance.

The responses were also analysed using a supervised machine learning tool, such as linear discriminant analysis (LDA), a statistical model that transforms multivariate data into a reduced number of variables through orthogonal linear combinations.^13, 14^ LDA showed five non-overlapping independent clusters corresponding to the five strains (**Figure S9b**). The quality of the LDA classifier was confirmed using leave-one-out cross-validation analysis. Jackknife analysis on the training set (5 strains × 8 replicates × 4 channels) revealed 100% between-group cross-validation accuracy, signifying that discriminant analysis is a robust tool for this sensing approach. The LDA used to discern the signatures was used as a training set, and a new set of sensor responses was used to predict the strains. In all cases, the sensor could accurately predict each strain (**Figure S9b** – pink dots). Taken together, we propose that the absorbance fingerprint resulting in the unique clustering of the different strains was due to surface characteristics that are influenced by virulence factors and sensitivity to antibiotics. The ability to stratify small changes demonstrated the ability of our sensor to ‘*smell’* subtle changes. This would mean that if an unknown strain is exposed to an array-based aptasensor, it can not only detect *S. aureus* but also predict strain features, such as the presence or absence of virulent genes and susceptibility to antibiotics.


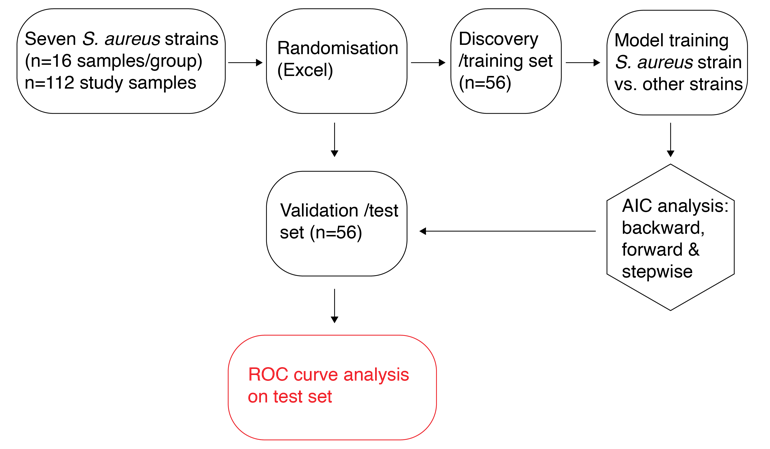


**Figure S10**. Workflow summary of the seven *S. aureus* strain study samples was randomised into a discovery/training set (n=56) and validation/test set (n=56) for training and testing of the aptamer performance.

To identify the key aptamer responsible for the identification of each strain, an Akaike Information Criterion Analysis was performed, as shown in the workflow summary (**Figure S10**). Based on this analysis, Aptamer 4 was a common key predictor for five *S. aureus* strains, while Aptamers 3, 2 and 1 were common predictors in four, three and one of the seven *S. aureus* strains, respectively (**Table 1**, main manuscript).

Sensing performance of array-based aptasensors in simulated wound fluid.

The robustness of the sensor array was tested, where the buffer was replaced with simulated wound fluid. The simulated fluid was prepared using 50% FBS and 50% maximum recovery diluent (containing 0.1% peptone and 0.9% sodium chloride w/v). Each experiment was carried out in replicates of eight.


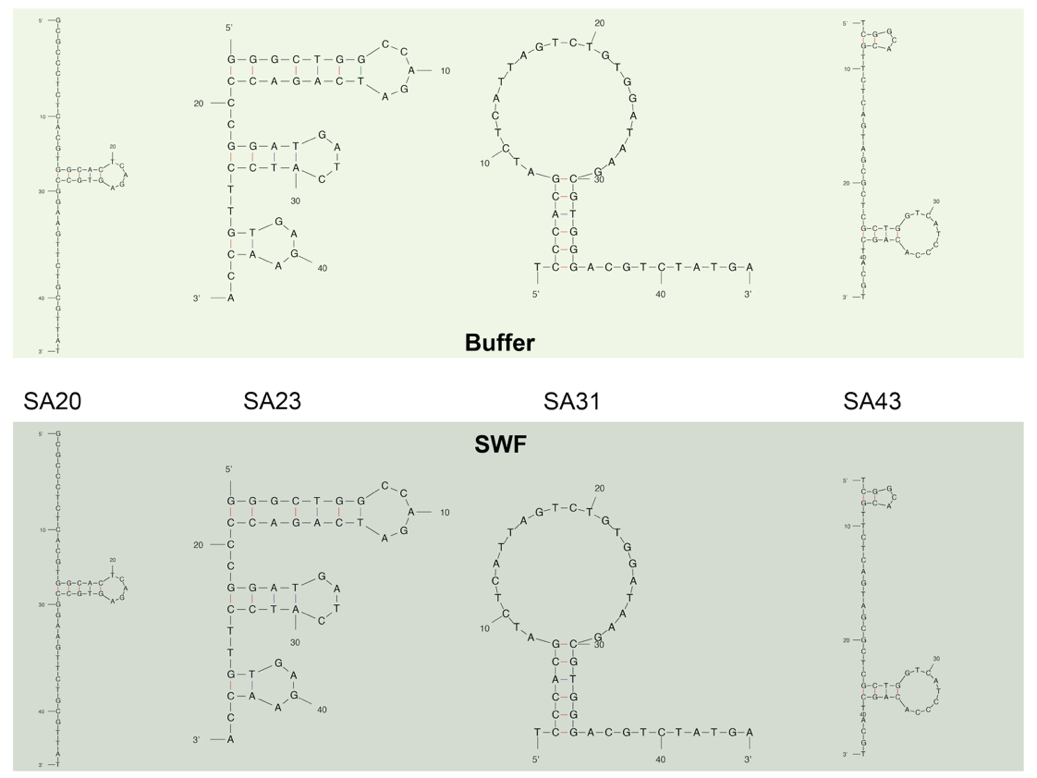


**Figure S11**. The predicted secondary structures of the four aptamers in buffer and SWF under ionic conditions similar to buffer and simulated wound fluid.

The colorimetric fingerprint for each strain (main manuscript, Figure 3a) was similar to that observed for the buffer (main manuscript, Figure 2a), with one notable exception. The response in the SWF was both more intense and rapid. To understand the origin of this enhanced response, we conducted complementary structural prediction^15^ and thermodynamic analyses^16^. MFold (UNAFold) analysis under ionic conditions similar to buffer and simulated wound fluid showed that the predicted secondary structure of all four aptamers (SA20, SA23, SA31, SA43) were conserved, suggesting that SWF does not induce a change in aptamer folding (**Figure S11**).

**Table S3.** Thermodynamic parameters obtained from mFold.

| Aptamer | Condition | ΔG (kcal/mole) | T_m_ (°C) | ΔH (kcal/mole) | ΔS (cal/(K·mole) | ΔΔG | ΔT_m_ |
| --- | --- | --- | --- | --- | --- | --- | --- |
| SA20 | Buffer | -4.38 | 60.9 | -61.2 | -183.2 |  |  |
|  | SWF | -4.52 | 61.7 | -61.2 | -182.7 | -0.14 | 0.8 |
| SA23 | Buffer | -2.93 | 45.5 | -109.7 | -344.2 |  |  |
|  | SWF | -3.32 | 46.6 | -109.7 | -342.9 | -0.39 | 1.1 |
| SA31 | Buffer | -4.54 | 58.9 | -68.6 | -206.5 |  |  |
|  | SWF | -4.73 | 59.9 | -68.6 | -205.9 | -0.19 | 1 |
| SA43 | Buffer | -2.93 | 51.5 | -65.5 | -201.7 |  |  |
|  | SWF | -3.13 | 52.5 | -65.5 | -201 | -0.2 | 1 |

The thermodynamic data revealed a consistent stabilisation of aptamer folding in SWF (**Table S3**). For all aptamers, the Gibbs free energy (ΔG) became more favourable in the presence of SWF (ΔΔG = ΔG_With SWF_ - ΔG_Without SWF_), accompanied by an increase in the melting temperature (See Table below). The enthalpy (ΔH) remained unchanged, indicating that the stabilisation arises from entropic and electrostatic effects rather than changes in base-pairing interactions. Given the small sample size (n = 4), both non-parametric (Wilcoxon) and parametric (paired t-test) statistical analyses were applied. The Wilcoxon signed-rank test did not reach statistical significance (p = 0.125), reflecting limited statistical power. However, the paired t-test indicated a significant shift in ΔG (p = 0.025). The effect size analysis showed a large stabilisation effect (paired Cohen’s d = -2.09), and the non-parametric bootstrap analysis (Bootstrap 95% Confidence Interval) estimated the median stabilisation to be between -0.39 and -0.14 kcal⋅mol^-1^ (95% CI), which does not cross zero. Taken together, these results demonstrate a directionally consistent stabilisation of aptamer folding in SWF. This stabilisation can be attributed to the high ionic strength of SWF, where counter-ions effectively shield the electrostatic repulsion between phosphate groups along the DNA backbone, lowering the free-energy barrier for formation of the folded state. The higher T_m_​ and more favourable ΔG in SWF suggest that a greater fraction of aptamers remains in the correct, binding-competent conformation at the assay temperature (37 °C). Therefore, by increasing the pool of correctly folded aptamers, the SWF matrix improves the potential for target binding and subsequently the signal generation, leading to increased sensitivity.

Clinical advantage and positioning.

To contextualise the clinical relevance of the proposed aptasensor platform, we compared its performance with commonly used diagnostic methods, including PCR, culture-based assays, and ELISA (**Table S4**). While PCR and culture methods remain the gold standard for clinical confirmation, they require specialised laboratories, trained personnel, and extended turnaround times. Our platform is designed as a rapid, low-cost, screening tool capable of strain-level discrimination without sequence information, predicting virulence markers such as PVL status, and recognising previously unencountered strains through pattern-recognition-based fingerprinting.

**Table S4**. Comparison of the proposed aptasensor array with conventional diagnostic methods for the detection of *S. aureus*.

| **Method** | **Limit of Detection (LOD)** | **Detection Time** | **Strain Differentiation** | **Virulence Marker Detection (e.g., PVL)** | **Cost per Test** | **Operational Complexity** | **Clinical Positioning** |
| --- | --- | --- | --- | --- | --- | --- | --- |
| **Culture Method** | ~10-100 CFU/mL | 24-72 h | Limited; requires additional biochemical assays | Requires additional PCR or immunoassays | Low-Moderate | Moderate; requires lab infrastructure | Gold-standard confirmation; slow but definitive |
| **PCR (including qPCR)** | 1-10 copies | 2-6 h | Limited; detects genes, not phenotypes | Yes (gene-based detection) | Moderate-High | High; requires skilled personnel and instrumentation | Rapid, high-sensitivity laboratory confirmation |
| **ELISA** | ng/mL levels (protein-based) | 2-5 h | No strain differentiation | Yes, depending on antibody | Moderate | Moderate; requires ELISA reader | Protein-level confirmation of specific markers |
| **Newly developed Apta-sensor Platform** | ~10^3^ CFU/mL | **<30 minutes** | Yes - strain-specific fingerprints (phenotyping) | **Yes - phenotypic inference (e.g., PVL)** | **Very low** | **Low; minimal instrumentation** | **Rapid screening; predictive capability for unencountered strains** |

**References**

(1) Crew, E.; Yan, H.; Lin, L.; Yin, J.; Skeete, Z.; Kotlyar, T.; Tchah, N.; Lee, J.; Bellavia, M.; Goodshaw, I. DNA assembly and enzymatic cutting in solutions: a gold nanoparticle based SERS detection strategy. *Analyst* **2013**, *138* (17), 4941–4949.

(2) Huang, S.; Xiang, H.; Lv, J.; Zhu, D.; Yu, L.; Guo, Y.; Xu, L. Au nanozyme-based colorimetric sensor array integrates machine learning to identify and discriminate monosaccharides. *Journal of Colloid and Interface Science* **2024**, *672*, 200–208. DOI: <https://doi.org/10.1016/j.jcis.2024.06.003>.

(3) Weerathunge, P.; Ramanathan, R.; Shukla, R.; Sharma, T. K.; Bansal, V. Aptamer-controlled reversible inhibition of gold nanozyme activity for pesticide sensing. *Anal. Chem.* **2014**, *86* (24), 11937–11941.

(4) Weerathunge, P.; Ramanathan, R.; Torok, V. A.; Hodgson, K.; Xu, Y.; Goodacre, R.; Behera, B. K.; Bansal, V. Ultrasensitive colorimetric detection of murine norovirus using NanoZyme aptasensor. *Anal. Chem.* **2019**, *91* (5), 3270–3276.

(5) Stefan, L.; Denat, F.; Monchaud, D. Insights into how nucleotide supplements enhance the peroxidase-mimicking DNAzyme activity of the G-quadruplex/hemin system. *Nucleic Acids Research* **2012**, *40* (17), 8759–8772. DOI: 10.1093/nar/gks581 PMC.

(6) Weerathunge, P.; Behera, B. K.; Zihara, S.; Singh, M.; Naveen Prasad, S.; Hashmi, S.; Mariathomas, P. R. D.; Bansal, V.; Ramanathan, R. Dynamic interactions between peroxidase-mimic silver NanoZymes and chlorpyrifos-specific aptamers enable highly-specific pesticide sensing in river water. *Analytica chimica acta* **2019**, *1083*, 157–165.

(7) Weerathunge, P.; Pooja, D.; Singh, M.; Kulhari, H.; Mayes, E. L.; Bansal, V.; Ramanathan, R. Transferrin-conjugated quasi-cubic SPIONs for cellular receptor profiling and detection of brain cancer. *Sens. Actuator. B.* **2019**, 126737.

(8) Wei, H.; Wang, E. Nanomaterials with enzyme-like characteristics (nanozymes): next-generation artificial enzymes. *Chem. Soc. Rev.* **2013**, *42* (14), 6060–6093, 10.1039/C3CS35486E. DOI: 10.1039/C3CS35486E.

(9) Wu, J.; Wang, X.; Wang, Q.; Lou, Z.; Li, S.; Zhu, Y.; Qin, L.; Wei, H. Nanomaterials with enzyme-like characteristics (nanozymes): Next-generation artificial enzymes (II). *Chem. Soc. Rev.* **2019**, *48* (4), 1004–1076, Review. DOI: 10.1039/c8cs00457a Scopus.

(10) Cao, X.; Li, S.; Chen, L.; Ding, H.; Xu, H.; Huang, Y.; Li, J.; Liu, N.; Cao, W.; Zhu, Y.; et al. Combining use of a panel of ssDNA aptamers in the detection of Staphylococcus aureus. *Nucleic Acids Res.* **2009**, *37* (14), 4621–4628. DOI: 10.1093/nar/gkp489 PMC.

(11) Yang, Y.; Chen, Z.; Pan, Y.; Zhang, Y.; Le, T. Interactions of metal-based nanozymes with aptamers, from the design of nanozyme to its application in aptasensor: Advances and perspectives. *Talanta* **2025**, *286*, 127450. DOI: <https://doi.org/10.1016/j.talanta.2024.127450>.

(12) Melles, D. C.; van Leeuwen, W. B.; Boelens, H. A. M.; Peeters, J. K.; Verbrugh, H. A.; van Belkum, A. Panton-Valentine leukocidin genes in Staphylococcus aureus. *Emerg. Infect. Dis.* **2006**, *12* (7), 1174–1175. DOI: 10.3201/eid1207.050865 PubMed.

(13) Saha, K.; Agasti, S. S.; Kim, C.; Li, X.; Rotello, V. M. Gold nanoparticles in chemical and biological sensing. *Chem. Rev.* **2012**, *112* (5), 2739–2779.

(14) Muhamadali, H.; Subaihi, A.; Mohammadtaheri, M.; Xu, Y.; Ellis, D. I.; Ramanathan, R.; Bansal, V.; Goodacre, R. Rapid, accurate, and comparative differentiation of clinically and industrially relevant microorganisms via multiple vibrational spectroscopic fingerprinting. *Analyst* **2016**, *141* (17), 5127–5136.

(15) Zuker, M. Mfold web server for nucleic acid folding and hybridization prediction. *Nucleic Acids Res.* **3003**, *31* (13), 3406–3415.

(16) SantaLucia Jr., J. A unified view of polymer, dumbbell, and oligonucleotide DNA nearest-neighbor thermodynamics. *Proc. Natl. Acad. Sci. USA* **1998**, *95*, 1460–1465.
